# DemaDb: an integrated dematiaceous fungal genomes database

**DOI:** 10.1093/database/baw008

**Published:** 2016-03-15

**Authors:** Chee Sian Kuan, Su Mei Yew, Chai Ling Chan, Yue Fen Toh, Kok Wei Lee, Wei-Hien Cheong, Wai-Yan Yee, Chee-Choong Hoh, Soon-Joo Yap, Kee Peng Ng

**Affiliations:** 1 Department of Medical Microbiology, Faculty of Medicine, University of Malaya, 50603 Kuala Lumpur, Malaysia; 2 Codon Genomics SB, No.26, Jalan Dutamas 7, Taman Dutamas, Balakong, 43200 Seri Kembangan, Selangor Darul Ehsan, Malaysia

## Abstract

Many species of dematiaceous fungi are associated with allergic reactions and potentially fatal diseases in human, especially in tropical climates. Over the past 10 years, we have isolated more than 400 dematiaceous fungi from various clinical samples. In this study, DemaDb, an integrated database was designed to support the integration and analysis of dematiaceous fungal genomes. A total of 92 072 putative genes and 6527 pathways that identified in eight dematiaceous fungi (*Bipolaris papendorfii* UM 226, *Daldinia eschscholtzii* UM 1400, *D. eschscholtzii* UM 1020, *Pyrenochaeta unguis-hominis* UM 256, *Ochroconis mirabilis* UM 578, *Cladosporium sphaerospermum* UM 843, *Herpotrichiellaceae* sp. UM 238 and *Pleosporales* sp. UM 1110) were deposited in DemaDb. DemaDb includes functional annotations for all predicted gene models in all genomes, such as Gene Ontology, EuKaryotic Orthologous Groups, Kyoto Encyclopedia of Genes and Genomes (KEGG), Pfam and InterProScan. All predicted protein models were further functionally annotated to Carbohydrate-Active enzymes, peptidases, secondary metabolites and virulence factors. DemaDb Genome Browser enables users to browse and visualize entire genomes with annotation data including gene prediction, structure, orientation and custom feature tracks. The Pathway Browser based on the KEGG pathway database allows users to look into molecular interaction and reaction networks for all KEGG annotated genes. The availability of downloadable files containing assembly, nucleic acid, as well as protein data allows the direct retrieval for further downstream works. DemaDb is a useful resource for fungal research community especially those involved in genome-scale analysis, functional genomics, genetics and disease studies of dematiaceous fungi.

**Database URL:** http://fungaldb.um.edu.my

## Introduction

The kingdom fungi is made up of large eukaryotic organisms consisting of more than 100 000 species, including unicellular yeasts and multicellular fungi known as moulds and mushrooms (1). Dematiaceous fungi (brown-pigmented) are a large and heterogeneous group of moulds that produce melanin pigment in the cell wall of fungal hyphae or the conidia (2). Dematiaceous fungi occupy a plethora of niches, being found in soil, wood, as well as associated with plants as endophytes, saprophytes, parasites or plant pathogens (3–5). Until 2008, >130 species from 70 genera of dematiaceous fungi have been implicated in a wide range of human diseases, such as eumycetoma, chromoblastomycosis and phaeohyphomycosis (6). *Alternaria* spp., *Bipolaris* spp., *Cladophialophora bantiana*, *Curvularia* spp., *Exophiala* spp., *Fonsecaea pedrosoi*, *Madurella* spp., *Scedosporium prolificans*, *Neoscytalidium dimidiatum* and *Wangiella dermatitidis* are among the most important human pathogens commonly found in the tropical and subtropical climates (2). Additional reported cases worldwide further expand the existing long list of potential pathogens.

From 2008 to 2015, we have isolated a total of 437 dematiaceous fungi in the Mycology Unit of University Malaya Medical Centre (UMMC), Malaysia. These clinical isolates were recovered from superficial skin samples, nails, subcutaneous tissues, and nasopharyngeal secretion, blood, and tissue biopsies (7). Among these isolates, one strain of *Pyrenochaeta unguis-hominis* and two strains of *Nigrospora oryzae* demonstrated potential multidrug resistance features. In addition, several of the isolates are rare human pathogens or non-reported human pathogens, such as *Bipolaris papendorfii* (8), *Daldinia eschscholtzii* (9), *Pyrenochaeta* sp. (10), *Ochroconis* sp. (11) and *Cladosporium sphaerospermum* (12). Phylogenetic relationship of these dematiaceous fungi has been described by Yew *et al.* (7). The internal transcribed spacer (ITS)-based phylogenetic analysis resolved them into four distinct classes of Dothideomycetes, Sordariomycetes, Eurotiomycetes and one unclassified cluster (7).

The rapid advancement of Next-Generation Sequencing technologies has led to the sequencing of many fungal genomes, paving the way to decipher their biology and the underlying mechanisms of fungal pathogenicity and multidrug resistance. Several web-based analyses are also available for annotation of genes predicted from high-throughput genomic data to gain insight into the fungal living system machinery (13). However, the true challenge is to integrate the multiple sources of genomics data into useful information (14). In this work, we design the DemaDb that enables mycologists to access easily and analyse the genomics data using a genome browser. Currently, a total of eight genomes (*B. papendorfii*, *D. eschscholtzii*, *P. unguis-hominis*, *C. sphaerospermum*, *Ochroconis* *mirabilis*, *Herpotrichiellaceae* sp. and *Pleosporales* sp.) have been integrated into the DemaDb. Considering that dematiaceous fungal genomes will be generated from future projects, it is essential to manage and integrate the data generated from different analyses in a more organized manner. The current version of DemaDb is freely available at fungaldb.um.edu.my and an improved version with additional genomes is forthcoming.

## Database Organization

**Figure 1.**
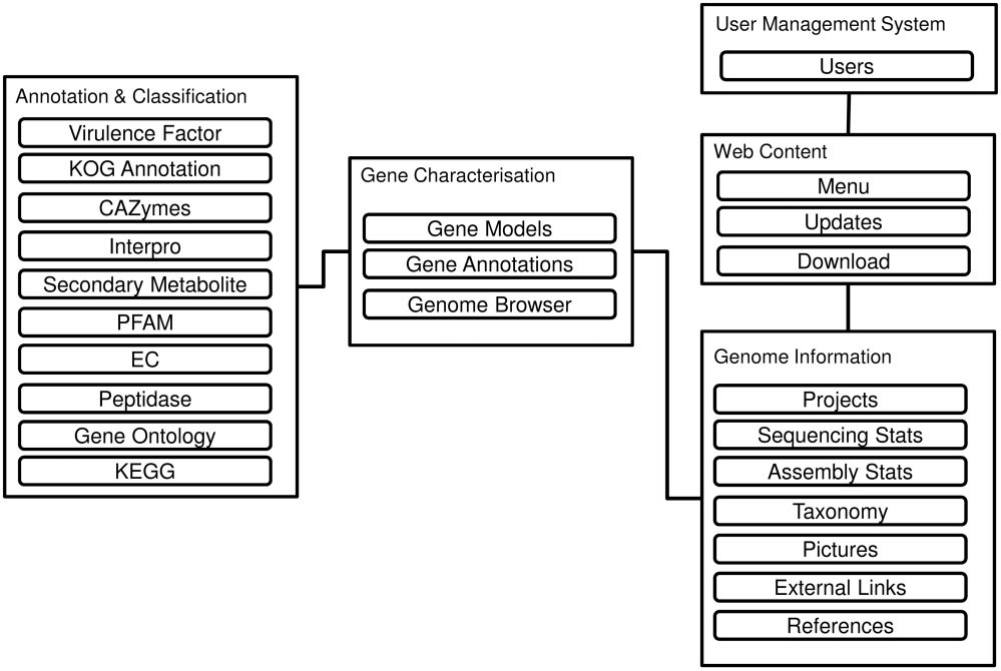
The database schema of DemaDb.

Figure 1 reveals the database schema of the DemaDb. DemaDb is built using a typical LAMP (Linux, Apache, MySQL and PHP) stack as the back-end components, along with Javascript and CSS (Cascading Style Sheet) for the front-end Web User Interface. The data are processed and stored in the MySQL relational database. The genome information is stored under ‘Genome Information’ table that records all the detailed information such as taxonomy classification, sequencing statistics, assembly statistics, pictures, references and external links. The information of gene models, annotations and genome browser links for each fungal genome is stored under ‘Gene characterization’ tables. ‘Annotation and Classification’ table is used to store various detail annotation, including Pfam, InterProScan, Gene Ontology (GO), EuKaryotic Orthologous Groups (KOG), Kyoto Encyclopedia of Genes and Genomes (KEGG), Carbohydrate-Active enzymes (CAZymes), peptidases, secondary metabolites and virulence factors.

## Usage and Utility of Basic Data

**Figure 2.**
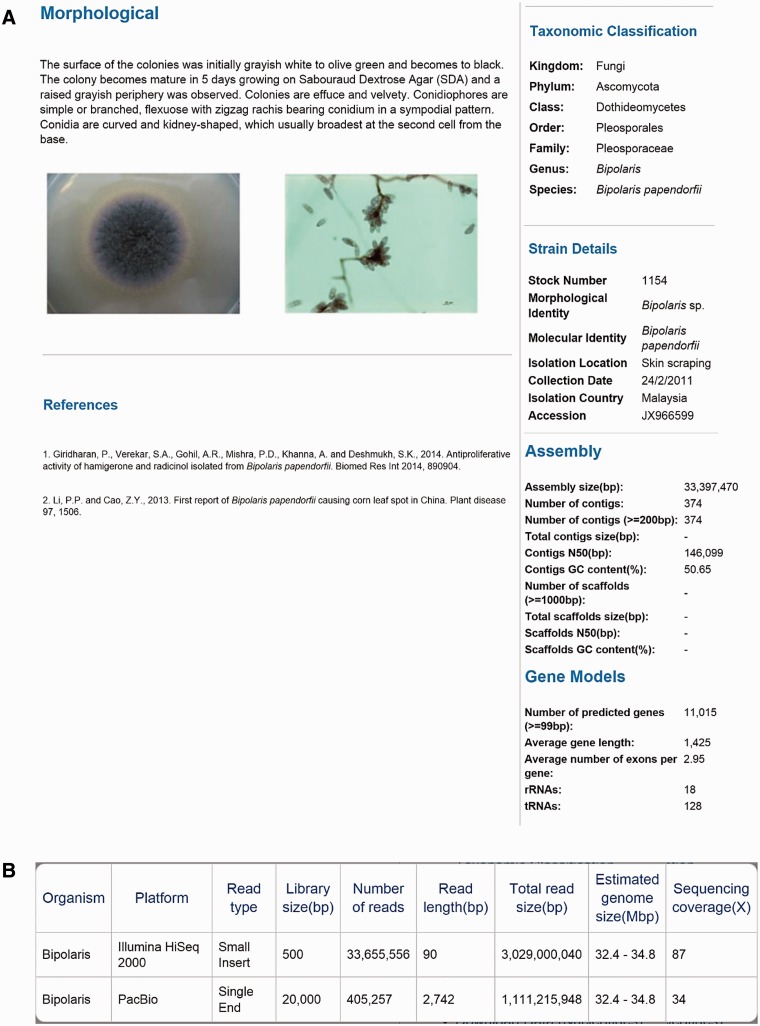
The layout of Project page for *B. papendorfii* UM 226 genome. (**A**) Morphological features (colonial characteristic and microscopic morphology) of *B. papendorfii* UM 226 are shown on the left, whereas taxonomic classification, strain details, assembly statistics and gene models are shown on the right. (**B**) In the Sequencing Stats tab, an overview of sequencing technology platform, read type, library size (bp), number of reads, read length (bp), total read size (bp), estimated genome size (Mbp) and sequencing coverage (×) are displayed.

A total of eight dematiaceous fungi that isolated from various samples (skin scraping, blood and nasopharyngeal secretion) were collected from University of Malaya between 2011 and 2014 (Table 1). The genomes of these rare and potential human pathogens were then sequenced and deposited in DemaDb. DemaDb is a web database that built based on a relational database system that contains dematiaceous fungal genomic profiles. The genome in DemaDb has its individual profile page with strain characteristics and genomic details. The information included on this page comprises the strain details, colonial characteristics on Sabouraud Dextrose Agar, microscopic morphology, taxonomic classification, assembly statistics and gene models (Figure 2A). The individual fungal profiles are linked to the NCBI taxonomic browser and The Catalogue of Life (15) to provide information on taxonomic hierarchy, distribution and ecological environment. Users can also explore the genome sequencing statistics in a single-genome context to gain insight into each sequencing platforms, read type, library size, genome size and sequencing coverage (Figure 2B). An overview of genome statistics (Table 2) and gene models (Table 3) of all genomes provides a basic comparative genomic analysis.

**Table 1.** Dematiaceous fungal genomes in the FungalDB

Fungal isolate	Isolation source	Isolation year
*Bipolaris papendorfii* UM 226	Skin scraping	2011
*Cladosporium sphaerospermum* UM 843	Blood	2008
*Daldinia eschscholtzii* UM 1400	Skin scraping	2012
*Daldinia eschscholtzii* UM 1020	Blood	2010
Herpotrichiellaceae sp. UM 238	Skin scraping	2011
*Ochroconis mirabilis* UM 578	Skin scraping	2012
Pleosporales sp. UM 1110	Nasopharyngeal secretion	2010
*Pyrenochaeta unguis-hominis* UM 256	Skin scraping	2011

**Table 2.** Comparative gene models. Number of predicted genes, average gene length (bp), average number of exon per gene, rRNAs, and tRNAs were shown in gene models page

Organism	Number of predicted genes (≥99 bp)	Average gene length (bp)	Average number of exons per gene	rRNAs	tRNAs
*Bipolaris papendorfii* UM 226	11 015	1425	2.95	18	128
*Cladosporium sphaerospermum* UM 843	9652	1482	2.26	42	196
*Daldinia eschscholtzii* UM 1400	10 822	1483	2.87	29	168
*Daldinia eschscholtzii* UM 1020	11 120	1616	2.82	28	156
*Herpotrichiellaceae* sp. UM 238	9409	1544	1.93	9	67
*Ochroconis mirabilis* UM 578	13 435	1411	2.57	14	71
*Pleosporales* sp. UM 1110	14 074	1512	2.59	25	101
*Pyrenochaeta unguis-hominis* UM 256	12 545	1517	2.76	33	121

**Table 3.** Comparative genomic statistics. Assembly size (bp), number of contigs, contigs N50, contigs GC content (%), number of scaffolds, scaffolds N50, and scaffolds GC content (%) were shown in genomic statistics page

Organism	Assembly size (bp)	Number of contigs	Number of contigs (≥200 bp)	Contigs N50 (bp)	Contigs GC content (%)	Number of scaffolds (≥1000 bp)	Scaffolds N50 (bp)	Scaffolds GC content (%)
*Bipolaris papendorfii* UM 226	33 397 470	374	374	146 099	50.65	–	–	–
*Cladosporium sphaerospermum* UM 843	26 892 198	877	867	92 815	55.67	155	969 659	55.32
*Daldinia eschscholtzii* UM 1400	35 760 939	1944	1939	33 562	46.8	104	701 334	46.51
*Daldinia eschscholtzii* UM 1020	35 494 957	644	644	112 742	46.81	598	114 605	46.80
*Herpotrichiellaceae* sp. UM 238	28 370 377	232	217	270 646	49.83	128	455 601	49.83
*Ochroconis mirabilis* UM 578	34 611 065	603	544	220 443	52.1	163	1 170 353	51.84
*Pleosporales* sp. UM 1110	36 912 818	500	498	308 776	51.14	419	312 067	51.14
*Pyrenochaeta unguis-hominis* UM 256	35 484 281	286	286	457 153	50.4	254	481 751	50.35

**Table 4.** Functional annotation for all predicted gene models. All predicted gene models were functionally annotated on the basis of KEGG, EC, GO KOG, Pfam, Interpro, CAZyme, secondary metabolite, peptidase, and virulence factor classification. Individual functional annotation from this track is linked to corresponding page for additional information

Organism	KEGG	EC	GO	KOG	PFAM	InterPro	CAZyme	CAZyme unique	Secondary metabolite	Peptidase	Peptidase unique	Virulence factor
*Bipolaris papendorfii*UM 226	1206	2381	7154	6296	–	8282	729	669	32	153	150	442
*Cladosporium sphaerospermum*UM 843	999	1817	6070	5853	7092	6842	605	566	16	136	134	405
*Daldinia eschscholtzii*UM 1400	975	1962	6471	6168	7690	7959	664	619	47	181	178	602
*Daldinia eschscholtzii*UM 1020	998	1883	6224	6195	–	7996	660	618	45	187	184	606
*Ochroconis mirabilis*UM 578	1012	2029	6829	6909	8923	9397	590	559	14	186	179	401
*Pyrenochaeta unguis-hominis*UM 256	1337	2555	7616	6813	–	9223	808	725	21	192	187	454


Gene ID	Family	Query start–end	Predicted active site residues	Predicted metal ligands	Hit name	Hit start–end	*E*-value	Merops annotation	Family type
									
UM256_gene_133	M35	218–352	E305	H304, H308, D317	MER001399	216–351	6.90e^−36^	Penicillolysin (*Penicillium citrinum*)	M35.001
UM256_gene_284	S12	38–405	S67, K70, Y174		MER179257	31–389	1.50e^−44^	Family S12 unassigned peptidases (*Aspergillus flavus*)	S12.UPW

## Uniform Functional Annotation

**Figure 3.**
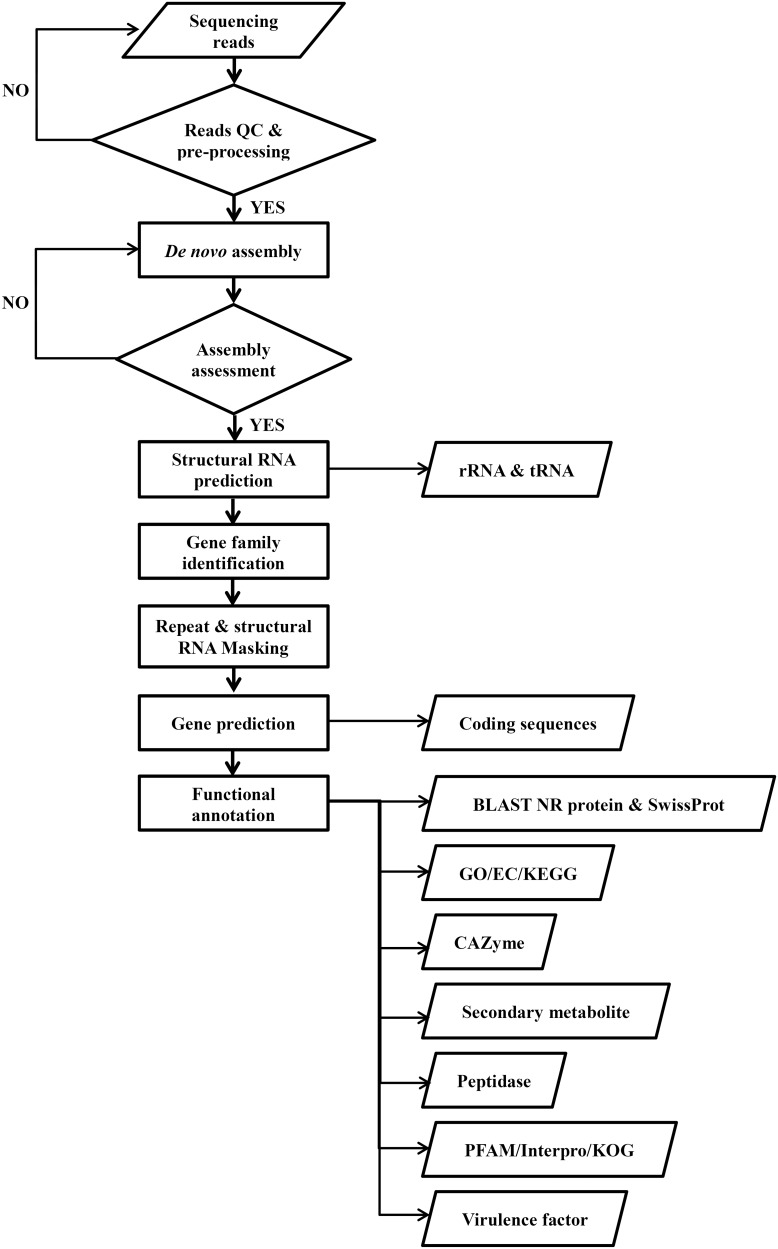
Workflow for the compilation of pipeline for all genomic data in DemaDb.

Application of the same annotation pipeline to all genomes is necessary for data integration in the DemaDb comparative genomic framework. All the raw data were pre-processed, assembled and functionally annotated using our pipeline. The compilation pipeline is provided in Figure 3. Protein-coding gene models were predicted from repeat-masked genome using GeneMark-ES version 2.3e (16). The annotation of protein-coding gene models was completed using BLAST (Basic Local Alignment Search Tool) alignments of fungal genomes against NCBI non-redundant (nr) protein and SwissProt databases. Individual rRNAs and tRNAs were identified using RNAmmer v1.2 (17) and tRNAscan-SE v1.3.1 (18), respectively. All putative proteins were then functionally annotated. Pfam protein families database (19) and InterproScan 5 (20) were used to identify functional domains and sites in all predicted protein models. GO and KEGG metabolic pathways matches were carried out using local BLAST2GO tools (21). All the predicted proteins were also ascribed to 21 different functional groups based on KOG (22) for additional functional interpretation. The CAZymes was annotated by submitting the predicted protein models to the databases of automated Carbohydrate-active enzyme ANnotation (dbCAN) (23). The peptidases were identified by mapping all protein models against MEROPS database (24). Genomic mapping of fungal secondary metabolite clusters was performed using web-based SMURF (Secondary Metabolite Unknown Regions Finder) (www.jcvi.org/smurf/) (25). The putative virulence factor was predicted using PHI-base (The Pathogen-Host Interaction Database) (26).

**Figure 4.**
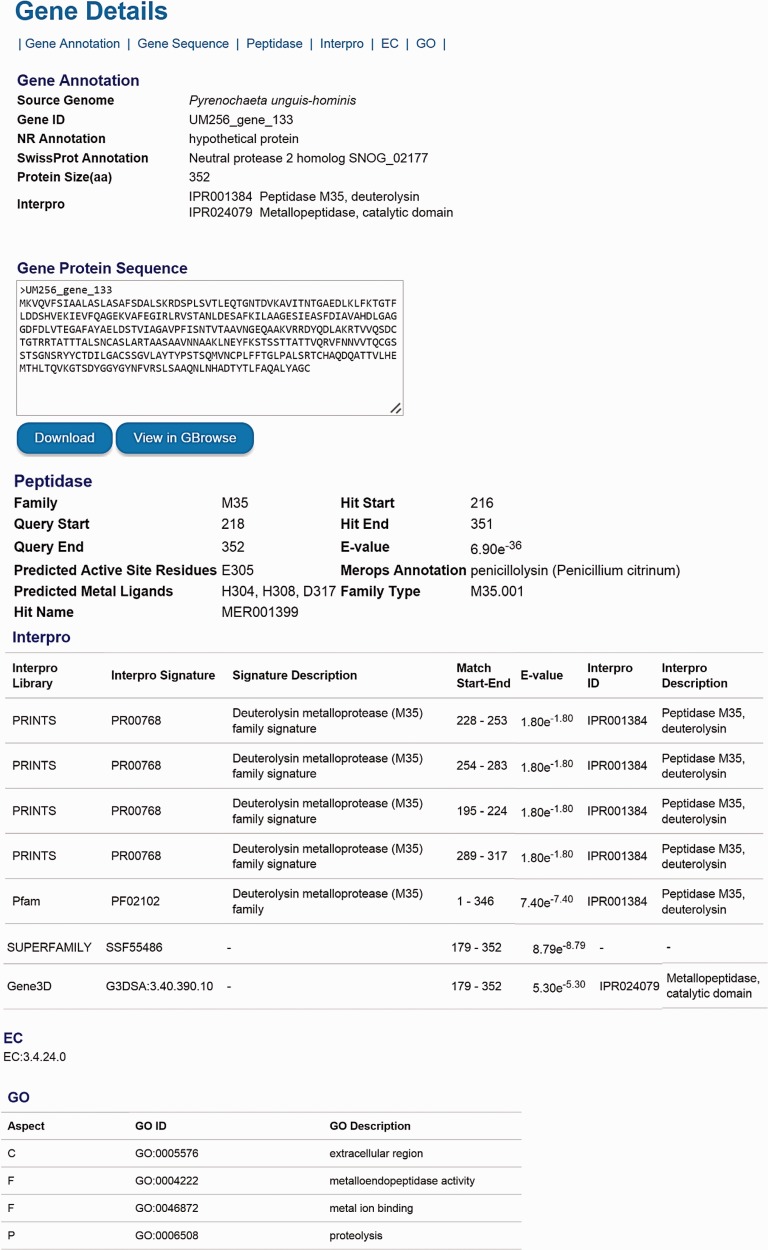
The layout of gene details page. The information, including gene ID, NCBI nr annotation, SwissProt annotation, GO annotation, protein size, domain sites, EC number and detailed functional annotation reports is available for every predicted gene.

The number of genes listed in the categories of KEGG, EC, GO, KOG, Pfam, Interpro, CAZyme, secondary metabolite, peptidase and virulence factor is available for comparison either among genomes in the DemaDb or other genomes outside the DemaDb (Tables 4). Each category is linked to a detailed annotation report for every predicted protein in the individual genome. For example, users can explore peptidase families, the range of peptidase, active site residues, ligands for catalytic metal ions and *E*-value for the match in all predicted peptidases (Tables 4). In-depth multidimensional analysis can be performed using these data to obtain clues about their fungal lifestyle, adaptability, mating development, mechanisms underlying pathogenicity and drugs resistance. The details for every predicted gene, including gene ID, NCBI nr annotation, SwissProt annotation, GO annotation, amino acid sequence, protein size, domain sites, EC number, as well as the detailed functional annotation reports are available in the DemaDb (Figure 4). Functional annotations for *Herpotrichiellaceae* sp. UM 238 and *Pleosporales* sp. UM 1110 are in progress and will be included in an updated version of DemaDb.

## Genome Browser

**Figure 5.**
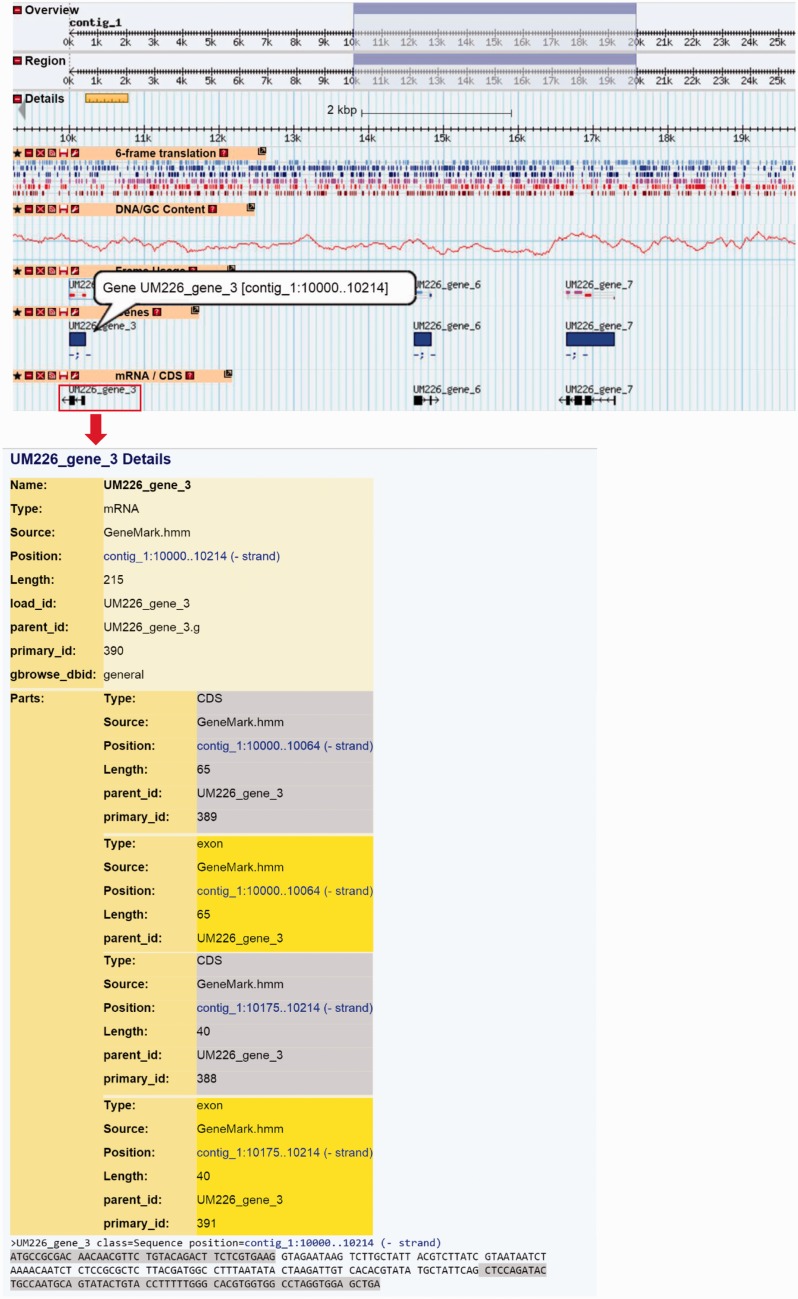
The layout of Genome Browser for *B. papendorfii* UM 226 genome. Horizontal tracks show genomic features of Contig 1. A highlighted blue rectangle indicates the genome region, that is, displayed in the details panel. A 6-frame translation track for DNA sequence is displayed in six different colour. The DNA/GC Content track represents the GC content in a given contig. In the genes track, blue bars indicate *B. papendorfii* UM 226 predicted genes. In the mRNA/CDs track, black arrows indicate *B. papendorfii* UM 226 mRNAs and their orientation. The information of a particular gene, including intron–exon organization, sequence and length is linked to each mRNA (black arrow).

Generic Genome Browser (GBrowse) developed by GMOD (27) is integrated into DemaDb to allow graphical web visualization of the genomic data. The DemaDb genome browser is accessible through the links at each genome project portal page and genome’s gene annotation & classification page, allowing the users to explore the genes of interest in a single-genome context. It also provides the navigation of the genomic regions for all eight fungal genomes in DemaDb, which can be freely switched through the drop-down menu of the data source box. Landmark or Region textbox acts as a universal search box, in which user could search by entering several types of inputs, including, but not limited to, range, gene name, chromosome name and description. It displays the predicted features of gene models along with their functional description (Figure 5). Single click on each gene track features will bring up a page showing additional annotation data retrieved from DemaDb database. By clicking on the gene ID, users will be brought to the detail description page of the annotation and amino acid sequence of the gene. The mRNA/CDS tracks are linked to additional gene details describing the gene structures and sequences (Figure 5). Additional tracks such as the DNA/GC Content track, 6-frame translation track and frame usage track can be displayed and configured using the toolbar.

## Pathway Browser

**Figure 6.**
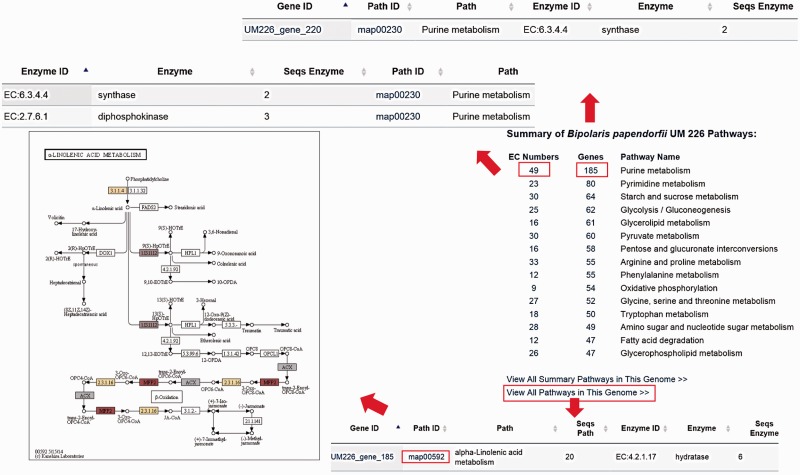
The layout of Pathway Browser for *B. papendorfii* UM 226 genome. The total number of genes and EC numbers for each pathway is displayed in the Pathway Browser page. Single click on each EC Number or gene will bring up a page showing additional information of genes that are involved in the specific pathway. Each KEGG annotated gene is also linked to a pathway map. The coloured EC numbers indicate that the genes are mapped to that EC number in the pathway.

The DemaDb Pathway Browser based on the KEGG pathway database enables users to analyse molecular interaction and reaction networks for all KEGG annotated genes. Pathway Browser provides a summary of metabolic pathways of each fungus and number of genes in each pathway. Users can select the pathways that they wish to analyse without the need for laborious search for all genes in every KEGG pathway. The top 15 hit pathways are shown in the Pathway Browser (Figure 6[TQ2]). Individual elements such as EC number and genes that are involved in specific pathway are linked to the corresponding pages for additional details. Users can also freely search for pathway map for all KEGG annotated genes (Figure 6).

## Downloadable Files

All assembly, nucleotides and proteins data can be downloaded in FASTA format to accommodate users who wish to pre-process the data based on additional parameters or conduct large-scale comparative genomic analysis.

## Future Perspective

DemaDb will be updated whenever a new genome and its detailed analysis are completed. Details for each release will be displayed in the homepage. Users are also encouraged to deposit and integrate their fungal genomes into DemaDb, which will be coordinated by the principal investigator of DemaDb. The users can send their assembled sequences (fasta format), predicted genes (fna format) or functional annotated proteins (faa format) directly to the principal investigator where the contact details are available in the ‘Contact’ section. The quality of the raw data will be accessed and reviewed, and, if approved, will be annotated and deposited into the DemaDb. Such collective works are important for comparative genomic analysis to gain a better understanding of dematiaceous fungi functional diversity and evolution.

Up to date, we have produced a total of 398 ITS sequences from various dematiaceous fungi recovered from different anatomical sites. This large set of data will be archived into an updated version of DemaDb. Apart from the ITS sequences, isolation source as well as macroscopic and microscopic characteristics of these isolates will be incorporated to provide comprehensive information of each isolate. Also, a taxonomic engine will be constructed to allow users to identify their fungal isolate by conducting a similarity search against our curated database, and an ITS-based phylogenetic tree will be generated for taxonomic classification. This extensive collection of data would serve as a referral point for fungi isolated in tropical countries, such as Malaysia. As some fungal species appeared to be geographically restricted (28), this would provide a glimpse of idea on the commonly encountered dematiaceous fungal pathogen in Malaysia.

To the best of our knowledge, there is no integrated view of dematiaceous fungal gene expression profiles obtained from RNA-sequencing (RNA-seq). Currently, we are performing RNA-seq of these clinical isolates. In the future, the DemaDb database will collect these RNA-seq data and use a standardized method to identify the gene expression levels. Integration of RNA-seq data into the genome browser will be one of the new features in the upcoming DemaDb database.

## Funding

This project was funded by University of Malaya and Ministry of Education, Malaysia under the High Impact Research MoE Grant UM.C/625/1/HIR/MOHE/MED/31 (No. H-20001-00-E000070)—principal investigator Professor Ng Kee Peng (University of Malaya). Funding for open access charge: High Impact Research MoE Grant UM.C/625/1/HIR/MOHE/MED/ 31 (No. H-20001-00-E000070).

*Conflict of interest*. None declared.
